# Distinct pattern of one-carbon metabolism, a nutrient-sensitive pathway, in invasive breast cancer: A metabolomic study

**DOI:** 10.18632/oncotarget.27575

**Published:** 2020-05-05

**Authors:** Jéssica Reis Santos, Dan Linetzky Waitzberg, Ismael Dale Cotrim Guerreiro da Silva, Tharcisio Citrangulo Tortelli Junior, Luciana Rodrigues Carvalho Barros, Gisele André Baptista Canuto, Andréa Tedesco Faccio, Lydia Fumiko Yamaguchi, Massuo Jorge Kato, Marina Franco Maggi Tavares, Ana Cristina Martinez, Ângela Flavia Logullo, Raquel Suzana M.M. Torrinhas, Graziela Ravacci

**Affiliations:** ^1^ Gastroenterology Department, University of São Paulo School of Medicine (FMUSP), São Paulo, Brazil; ^2^ Gynecology Department, College of Medicine of the Federal University of São Paulo (EPM-UNIFESP), São Paulo, Brazil; ^3^ Centro de Investigação Translacional em Oncologia (LIM24), Departamento de Radiologia e Oncologia, Faculdade de Medicina da Universidade de São Paulo and Instituto do Câncer do Estado de São Paulo, São Paulo, Brazil; ^4^ Departamento de Química Fundamental, Instituto de Química, Universidade de São Paulo, São Paulo, Brazil; ^5^ Departamento de Química Analítica, Instituto de Química, Universidade Federal da Bahia, Salvador, Brazil

**Keywords:** invasive ductal carcinoma, targeted metabolomics, untargeted metabolomics, tumor metabolism, one-carbon metabolism

## Abstract

Altered cell metabolism is a hallmark of cancer and critical for its development. Particularly, activation of one-carbon metabolism in tumor cells can sustain oncogenesis while contributing to epigenetic changes and metabolic adaptation during tumor progression. We assessed whether increased one-carbon metabolism activity is a metabolic feature of invasive ductal carcinoma (IDC). Differences in the metabolic profile between biopsies from IDC (*n* = 47) and its adjacent tissue (*n* = 43) and between biopsies from different breast cancer subtypes were assessed by gas spectrometry in targeted (Biocrates Life Science^**®**^) and untargeted approaches, respectively. The metabolomics data were statistically treated using MetaboAnalyst 4.0, SIMCA P+ (version 12.01), Statistica 10 software and t test with *p* < 0.05. The Cancer Genome Atlas breast cancer dataset was also assessed to validate the metabolomic profile of IDC. Our targeted metabolomics analysis showed distinct metabolomics profiles between IDC and adjacent tissue, where IDC displayed a comparative enrichment of metabolites involved in one-carbon metabolism (serine, glycine, threonine, and methionine) and a predicted increase in the activity of pathways that receive and donate carbon units (i.e., folate, methionine, and homocysteine). In addition, the targeted and untargeted metabolomics analyses showed similar metabolomics profiles between breast cancer subtypes. The gene set enrichment analysis identified different transcription-related functions between IDC and non-tumor tissues that involved one-carbon metabolism. Our data suggest that one-carbon metabolism may be a central pathway in IDC and even in general breast tumors, representing a potential target for its treatment and prevention.

## INTRODUCTION

Breast cancer is the most common malignancy in women and the leading cause of their cancer-related deaths, with an estimated 23% prevalence and 14% mortality rate worldwide [1, 2]. The invasive ductal carcinoma (IDC) accounts for 75% of reported breast cancer cases. This breast tumor usually rises from lobular and ductal epithelial cells at the terminal duct lobular unit, comprising different subtypes based on histological and molecular features [3–5].

Different breast cancer subtypes (i.e., luminal A, luminal B, HER2 enriched, and basal-like) reflect the disease heterogeneity [3, 5–7]. These are defined by genetic changes that result in the overexpression of oncogenes and downregulation of tumor suppressor genes, generating different malignant phenotypes [8–10]. Several oncogenes (i. e., RAS, PI3K, TP53 and MYC) can regulate metabolic pathways that are critical for cell survival in the inhospitable tumor microenvironment, where oxygen and nutrients sources are highly limited [11, 12].

Accumulating evidence has highlighted that cancer cells differ from their normal counterparts in nutrient use, biomolecule synthesis and energy generation. In the last decade, distinct metabolic patterns between normal and cancer cells were recognized as a hallmark of the disease [13]. Screening for metabolic signatures of tumor cells by “omics” platforms has substantially contributed to a better understanding of cancer metabolism and behavior [14, 15].

Targeted or non-targeted metabolomics refers to the analysis of the metabolite pool of a living system (the metabolome). Thus, identifying metabolic changes using metabolomics may have a potential impact on the understanding and treatment of cancer [16].

One-carbon metabolism is activated in some cancers and can provide the building blocks and reducing power required to maintain high cell proliferation rates, a key feature of oncogenesis [17]. This pathway encompasses a broad range of biosynthetic reactions in both cytoplasm and mitochondria that catabolize reactions in different carbon sources to derive one-carbon (methyl) units, integrating several nutrients during this process. The generated carbon units can sustain fundamental cellular functions, including cellular biosynthesis, redox homeostasis and the epigenetic state [17, 18].

Mutations in TP53 and MYC, which are common in breast tumors, seem to increase one-carbon metabolism activity [19–23]. Aiming to contribute to the understanding of breast carcinogenesis and the identification of potential treatment targets, here we assessed whether the increased activity of one-carbon metabolism is a metabolic feature of breast cancers and whether it may represent a potential target for its treatment and prevention.

## RESULTS

### Targeted metabolomics profile of IDC and non-tumor adjacent breast tissue samples from breast cancer patients

The first aim of this work was to analyze metabolomic differences, mainly in metabolites associated with one-carbon metabolism, by applying a targeted metabolomics approach that considered only low-molecular-weight (m/z < 1500) ionizable molecules present in at least 50% of samples from each group. We searched for comparative differences in the metabolite profiles of IDC and non-tumor adjacent breast tissues.

**Figure 1:**
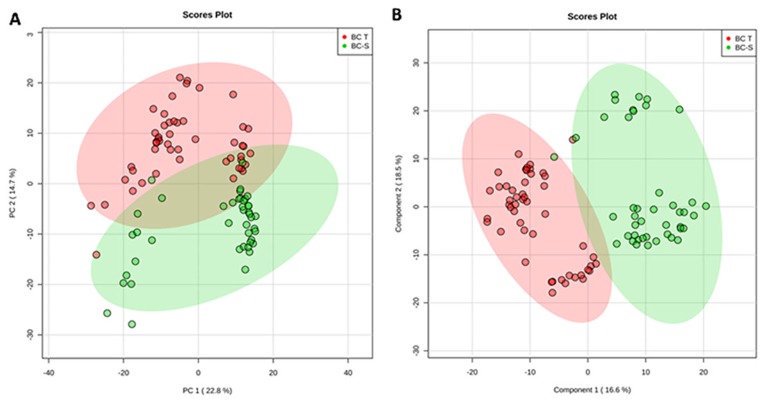
Tissue metabolomics multivariate analysis. BC-T: IDC; BC-S: non-tumor adjacent breast tissue. (**A**) Principal component analysis (PCA) showing the natural separation between breast tumor and non-tumor adjacent breast tissues. (**B**) Partial least square discriminant analysis (PLS-DA) showing robust separation among the groups (BC-T *vs* BC-S). Each point in the plot corresponds to a tissue sample.

For this purpose, we applied an unsupervised principal component analysis (PCA) that showed strong group separation between the two groups (Figure 1A), suggesting a specific metabolomics signature for each condition. This was further confirmed by applying partial least square discriminant analysis (PLS-DA - Figure 1B), which demonstrated robust group separation between groups and displayed good cross-validation results (max components = 5; C-V method = 10-fold CV; performance measure = Q2 - Supplementary Figure 1).

**Figure 2:**
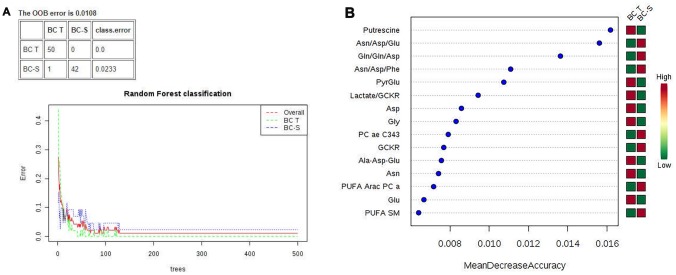
Random forest (RF) metabolite classification and its variables of importance in IDC and non-tumor adjacent breast tissues. BC-T: IDC; BC-S: non-tumor adjacent breast tissue. (**A**) Supervised class prediction analysis comparing BC-T and BC-S, with a 0.0108 overall classification error (0.0 for tumor tissue and 0.0233 for adjacent tissue); (**B**) Metabolites showing the most differentiation between BC-T and BC-S were selected from the RF and T test, in which the color code indicates green for low and red for high. BC-T: invasive ductal carcinoma - IDC | BC-S: non-tumor adjacent breast tissue. Asn/Asp/Glu: asparagine, aspartate, glutamate ratio; Gln/gln/asp: glutamine, glutamine, aspartate ratio; Asn/Asp/Phe: asparagine, aspartate, phenylalanine ratio; PyrGlu: pyruvate glutamate; GCKR: sum of hexoses; lactate/GCKR: lactate GCKR ratio; Gly: glycine; PC ae C34:3: phosphatidylcholine ae C34:4; Ala-Asp-Glu, sum alanine + aspartate + glutamine; PUFA ARAC PC aa: phosphatidylcholine enriched with arachidonic acid; PUFA SM: sphingomyelin enriched with *polyunsaturated fatty acid.*

Multivariate classification analyses were complemented by applying random forest (RF) analyses, a supervision class prediction model, to a) determine the capacity for a metabolomics profile to accurately classify tissue samples into their respective groups and b) identify the most important metabolites for class prediction and, hence, the strongest correlation with the respective disease. RF analysis from the metabolic profiles accurately classified almost 100% of the samples between their respective IDC and non-tumor adjacent breast tissue groups, with a 0.0108 out-of-bag (OOB) class error for both groups (Figure 2A). The major metabolites contributing to the classification are shown in Figure 2B.

### Altered metabolites

**Figure 3:**
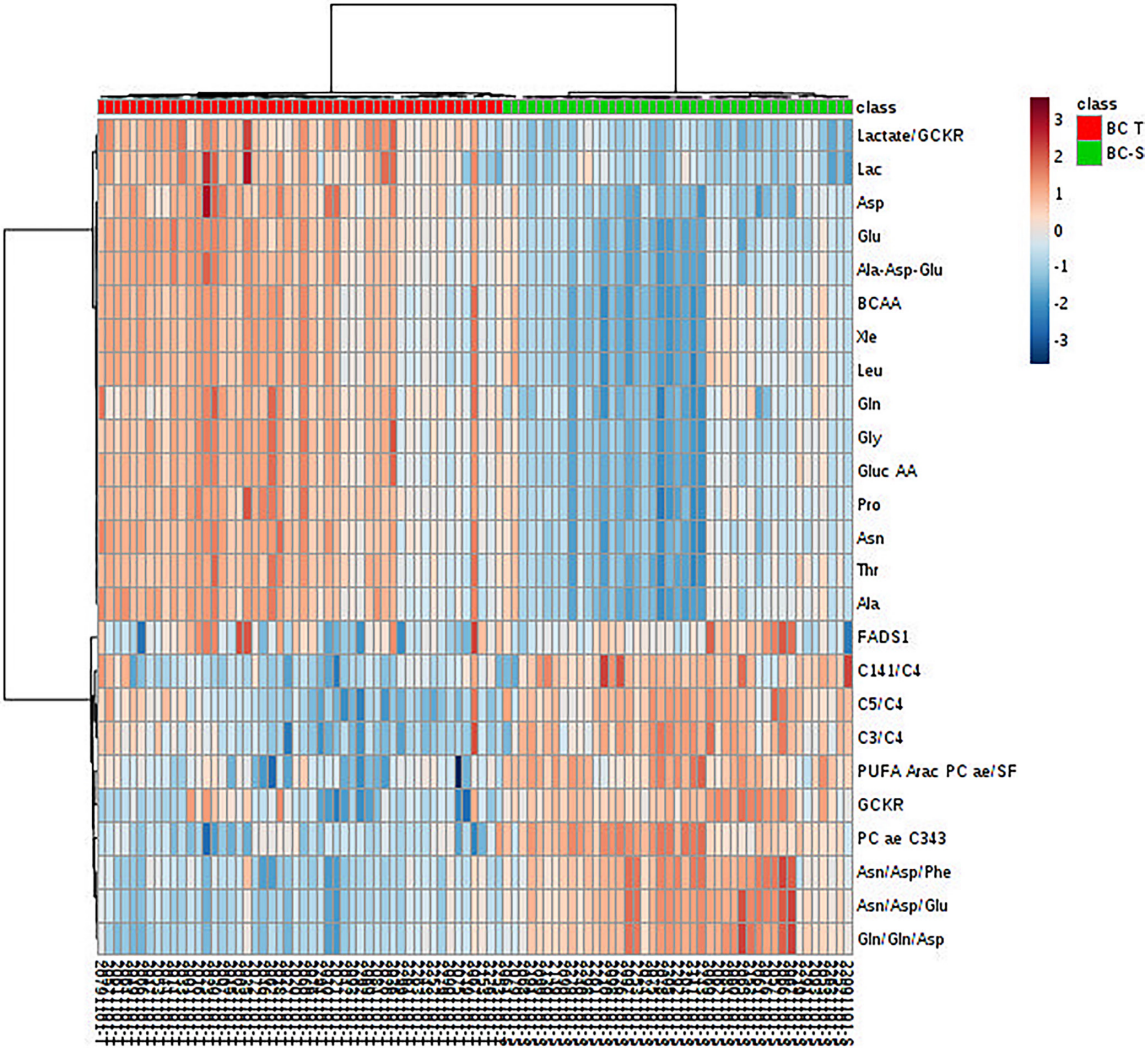
Metabolite hierarchical clustering in IDC and non-tumor adjacent breast tissues. BC-T: IDC; BC-S: non-tumor adjacent breast tissue. Horizontal columns: relative concentration of each biologically relevant metabolite displaying distinct metabolic patterns between BC-T and BC-S. Each bar in the horizontal columns represents the expression intensity. For example, the blue scale indicates a decreased level, while the red scale indicates an increased level. The dendrogram on the left was codirected based on the metabolite intensity expression profiles. GCKR: sum of hexoses; lactate/GCKR: lactate, glucose ratio; Lac: lactate; Asp: aspartate, Glu: glutamine; Ala-Asp-Glu: sum alanine + aspartate + glutamine; BCAA: branched-chain amino acid; Xle: sum leucine + isoleucine; Leu: leucine; Gln: glutamine; Gly: glycine; Gluc AA: gluconeogenic amino acids; Pro: proline; Asn: asparagine; Thr: threonine; Ala: alanine; FADS1: desaturated fatty acid (indirect analysis); C14:1/C4: tetradecanoylcarnitine, butyrylcarnitine ratio; C5/C4: valerylcarnitine, butyrylcarnitine ratio; C3/C4: propionyl, butyrylcarnitine ratio; PUFA ARAC PC ae/SFA: phosphatidylcholine enriched with arachidonic acid fatty acid and saturated fatty acid ratio; PC aa C34:3: phosphatidylcholine ae C34:3; Asn/Asp/Phe: asparagine, aspartate, phenylalanine ratio; Asn/Asp/Glu: asparagine, aspartate, glutamine ratio; Gln/gln/asp: glutamine, glutamine, aspartate ratio.

After multivariate statistics, the Student’s T-test was applied to search for metabolites that were significantly different between the IDC and non-tumor adjacent tissues (*p* < 0.05, Benjamini-Hochberg false discovery rate) and highlighted 99 metabolites with a significant difference in concentration between the two groups. In some of these metabolites, it was possible to apply a fold change (FC) analysis to determine the changes included in the groups (Supplementary Table 1). We further observed, by hierarchical analysis (multivariate statistics), whether these molecules could define the IDC metabolic status based on their individual relative concentration (Figure 3). All metabolic analyses were validated using software R. The results are available in Supplementary Material 3.

**Figure 4:**
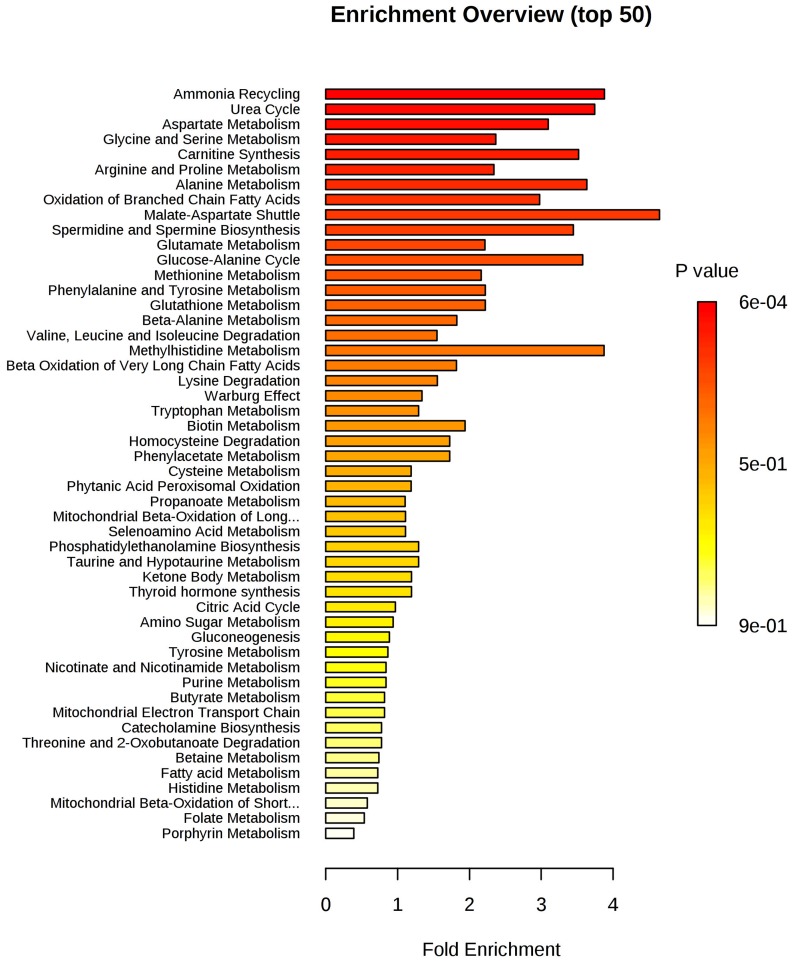
Metabolite set enrichment analysis (MSEA) of IDC. The horizontal bar graph summarizes 50 metabolic pathways using metabolites that were significantly altered in IDC.

**Figure 5:**
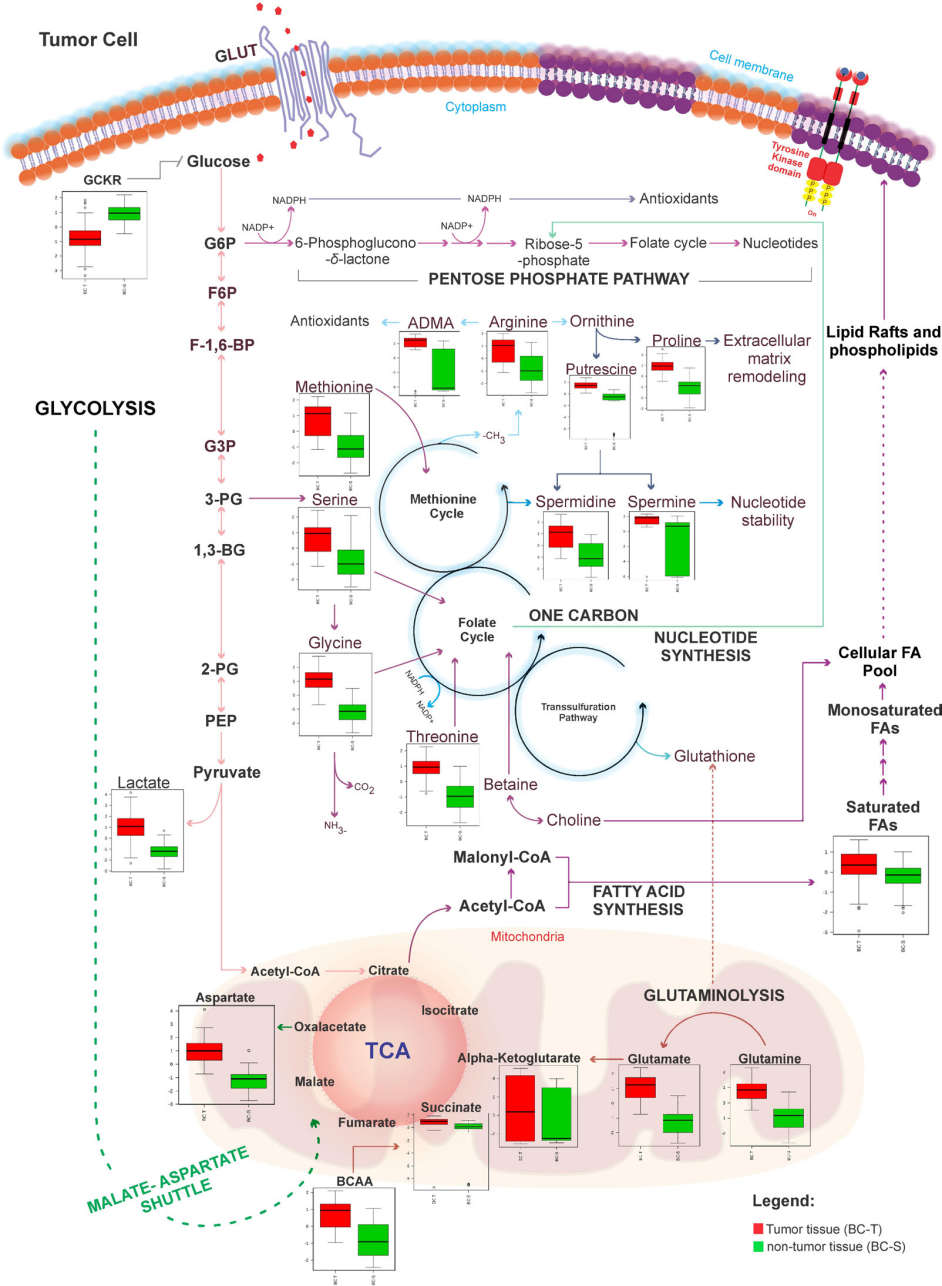
Metabolic network map between IDC and non-tumor adjacent breast tissues according to the main metabolites identified in this study. BC-T: IDC; BC-S: non-tumor adjacent breast tissue. Box plots: red, normalized concentration of the metabolite in BC-T. Box plots: green, normalized concentration of the metabolite in BC-S. In one-carbon metabolism, carbon unit generation into different cellular outputs involves a complex containing three pathways: the folate cycle, the methionine cycle, and the transsulfuration pathway. Compared with non-tumor breast adjacent tissue, metabolites that fuel one-carbon metabolism (serine, glycine, threonine, and methionine), in addition to classical pathways previously described in cancer (i. e., glycolysis, glutaminolysis, fatty acid metabolism), were all increased in IDC. These metabolites seem to work together to meet most requirements of metabolic pathways involved in cell proliferation, including cellular biosynthesis (nucleotide and amino acid synthesis, and membrane formation), maintenance of the genome (through the nucleotide pool), epigenetic regulation (methylation), and regulation of the redox state. GCKR: sum of hexoses; G6P: glucose 6-phosphate; F6P: fructose 6-phosphate; F-1,6BP: fructose 1,6-bisphosphate; G3P: glyceraldehyde 3-phosphate; 3-PG: 3-phosphoglycerate; 1-3 BG: 1,3-bisphosphoglycerate; 2-PG: 2-phosphoglycerate; PEP: phosphoenolpyruvic acid; ADMA: asymmetric dimethylarginine; NO: nitric oxide; ROS: reactive oxygen species; BCAA: branched-chain amino acid.

The set of metabolites that were significantly different between the groups was subjected to pathway enrichment analysis to elucidate the metabolic pathways that were perturbed in IDC (Figure 4). The top 15 pathways enriched in these tumor samples all involved one-carbon metabolism: ammonia recycling, urea cycle, aspartate metabolism, glycine and serine metabolism, carnitine synthesis, arginine and proline, alanine metabolism, oxidation of branched-chain fatty acids, malate-aspartate shuttle, spermidine and spermine biosynthesis, glutamate metabolism, glucose-alanine cycle, methionine metabolism, phenylalanine and tyrosine metabolism, and glutathione metabolism. A detailed analysis including all 55 identified pathways is provided in Supplementary Table 2, and the metabolic pathway network of the significantly altered metabolites in IDC is presented in Figure 5.

### Targeted metabolomics profile of breast cancer subtypes

After exploring the metabolomic changes in IDC, our next step was to check if these changes were independent of the IDC subtype, as described in Supplementary Material 1 - Supplementary Table 3, especially changes associated with one-carbon metabolism.

**Figure 6:**
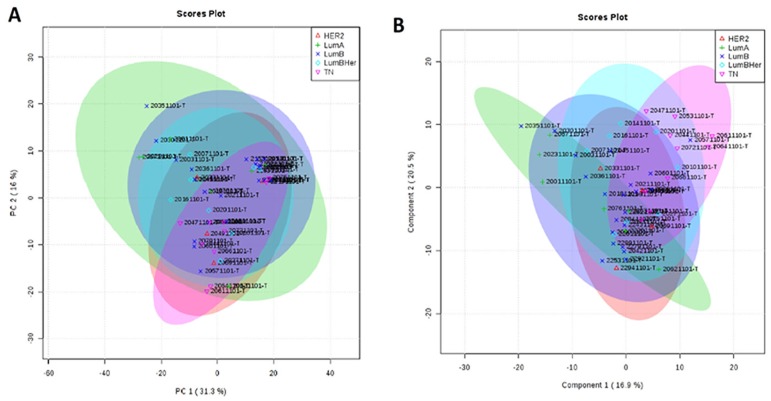
Metabolic targeted multivariate analysis. Red: Her-2; green: luminal A; purple: luminal B; blue: luminal B Her; pink, triple negative. (**A**) Principal component analysis (PCA) showing that was not possible to identify metabolic differences between breast cancer subtypes (**B**) Partial least square discriminant analysis (PLS-DA) showing that there was no metabolic separation between tumor subtypes.

According to our results, it was not possible to identify metabolic differences between breast cancer subtypes (Figure 6). After that, we performed a metabolic validation through an untargeted approach in a new cohort of breast cancer subtypes to verify if its behavior was common to subtypes.

### Untargeted metabolomics profile of breast cancer subtypes

**Figure 7:**
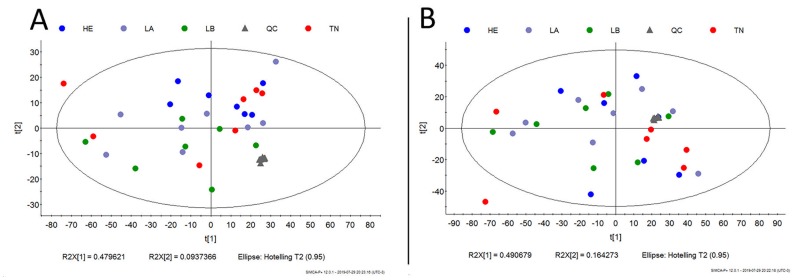
Metabolic untargeted multivariate analysis blue: Her-2; purple: luminal A; green: luminal B; gray: quality control; red: triple negative. (**A**) Principal component analysis (PCA) model R^2^ parameters = 0.787 and Q^2^ = 0.671. It was not possible to observe a natural separation between samples of breast cancer subtypes. (**B**) Partial least square discriminant analysis (PLS-DA) model R^2^ parameters = 0.771 and Q^2^ = 0.065. Model built with all data, without QC prediction. Evidence that there is no metabolic separation between tumor subtypes.

Our next step was to compare the untargeted metabolomics profile of different human breast tumor subtypes to examine whether there was a single metabolomics signature for this heterogeneous disease. The tumor samples (*n =* 42) were obtained from the AC Camargo Cancer Center biobank, and their metabolomics profiles were assessed through an untargeted approach by LC-MS analysis (global metabolomics profile). Principal component analysis (PCA) and partial least squares discriminant analysis (PLS-DA) did not show a relevant metabolic separation (Figure 7). The raw data for this analysis are available in Supplementary Material 2.

Both by targeted and untargeted approaches, the metabolic evaluation among breast cancer subtypes was similar, suggesting that the metabolic changes presented by the IDC may be a universal characteristic of the disease. In this scenario, our next step was to perform a genetic validation between tumor and non-tumor samples.

### Functional enrichment analysis of differentially expressed genes - validation cohort

**Figure 8:**
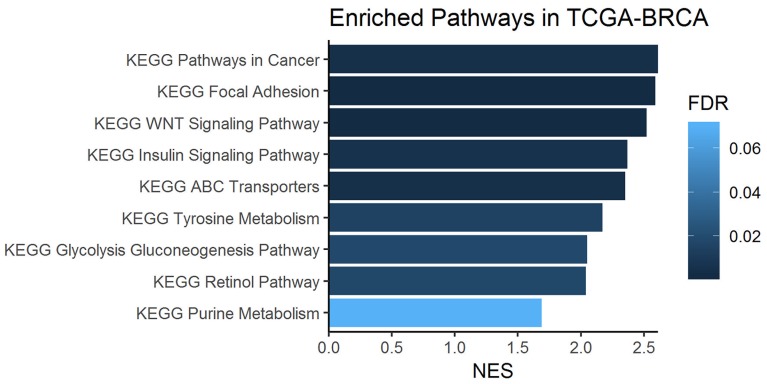
Enriched one-carbon related pathways in breast cancer samples from the KEGG database. Gene set enrichment analysis (GSEA) was performed on metabolism-related genes using the TCGA-BRCA cohort of tumors and normal tissue sample RNA-Seq data.

To assess whether the upregulation of one-carbon metabolism may be a general feature of IDC and whether this could be due to gene alterations, we performed a differential gene expression analysis comparing tumor and non-tumor samples from an independent patient cohort using the Cancer Genome Atlas breast cancer dataset (TCGA-BRCA) [24]. Only IDC samples from TCGA were included in this analysis. In Table 1, we highlight 10 processes that were biologically capable of differentiating breast cancer and normal tissue. Among them, we observed genes involved in one-carbon metabolism, specifically highlighted in Table 2, for the metabolic process *GO: 0008152. We then performed gene set enrichment analysis (GSEA) (Figure 8) [25].

**Table 1:** Genetic validation analysis performed in the cancer genome atlas (TCGA)

term_id	proteins	hits	*P* value	*P* value_fdr	term_description
**GO:0009987**	111	111	3E-254	7,6801E-251	cellular process
**GO:0008150**	108	108	1,3652E-246	1,7475E-243	biological process
**GO:0044763**	104	104	1,7493E-236	1,4927E-233	single-organism cellular process
**GO:0044699**	102	102	1,7975E-231	1,1504E-228	single-organism process
**GO:0065007**	99	99	5,2756E-224	2,7011E-221	biological regulation
**GO:0050789**	93	93	3,0673E-209	1,3087E-206	regulation of biological process
**GO:0050794**	91	91	2,2922E-204	8,383E-202	regulation of cellular process
*GO:0008152	75	75	3,7092E-166	1,1869E-163	metabolic process
**GO:0032501**	73	73	1,8012E-161	5,1235E-159	multicellular organismal process
**GO:0050896**	71	71	8,3822E-157	2,1459E-154	response to stimulus

^*^TCGA analysis was performed to identify biological functions capable of differentiating breast cancer and normal tissue.

**Table 2:** Genes involved in one-carbon metabolism identified through Gene Ontology (GO) by genetic validation

HGNC. symbol	Fold Change	Cellular Location
**ALDH1L1**	4.8561047775901	cytoplasm
**SHMT2**	–1.0479550141666	mitochondria
**MTHFD2**	–1.57882401191097	mitochondria
**TYMS**	–2.14173789433925	cytoplasm
**GLDC**	–3.13552794835523	mitochondria

## DISCUSSION

Aiming to contribute to data on the regulation of biological pathways underlying the malignant transformation of IDC, we assessed the metabolic profiles of human biopsies from primary tumors and their adjacent tissues. Our analysis displayed distinct metabolomic patterns with almost no overlap between IDC and adjacent tissue. A predominance of one-carbon metabolism markers characterized the tumor metabolic phenotype, as further supported by genomic data from external samples. Furthermore, similar metabolomics profiles between distinct IDC and breast cancers subtypes were also observed. Our findings suggest that one-carbon metabolism is involved in IDC carcinogenesis and may be a therapeutic molecular target, irrespective of its subtype. They also point out that this metabolic alteration may be a universal feature of breast cancers, but this hypothesis should be confirmed in the future.

It has been reported that approximately 10% of 3-PG are diverted from the glycolytic pathway to one-carbon metabolism by subsets of tumor cells (including breast tumors) and used in the *de novo* biosynthesis of serine (SSP), its main carbon donor [22, 23, 26]. Accordingly, in our metabolomics analysis, we observed a decreased sum of hexoses (GCKR) and increased serine and lactate levels in IDC samples (*vs*. adjacent tissue), suggesting that glycolysis may participate in the *de novo* synthesis of serine in this breast tumor.

Furthermore, our gene set enrichment analysis highlighted the activation of glycolysis-gluconeogenesis and insulin signaling pathways. Gluconeogenesis can contribute up to 70% of the total serine synthesized under high metabolic demand, as observed during carcinogenesis [19, 27]. Insulin is a major regulator of cell metabolism and regulates the translocation of glucose transporter 4 (GLUT4) to the plasma membrane, a process that initiates cellular glucose uptake (glycolysis), and overexpression of its receptors is often observed in tumor cells and associated with poor survival in patients with breast cancer [28]. Taken together, our data suggest an important role of these pathways and their importance for the metabolic plasticity of IDC by providing the main substrate for one-carbon metabolism activation.

It is worth noting that increased lactate levels generated from glycolysis contribute to extracellular environment acidification, which can promote the immune evasion of tumor cells [29] and is considered an important mechanism by which anaerobic glycolysis (Warburg effect) contributes to cancer aggressiveness [30]. An increased Warburg effect was also observed in the pathway enrichment analysis of our tumor samples (*vs*. adjacent tissue), in parallel to the indirect increase in β-alanine metabolism. Once β-alanine can be metabolized to carnosine (an intracellular buffer) [31], these findings suggest a neoplastic self-regulatory mechanism against excessive acidification and a negative glycolysis feedback mechanism in the IDC.

In addition to serine, there are alternative input pathways for one-carbon metabolism. An enzymatic glycine cleavage system that produces ammonia (NH_3_), carbon dioxide (CO_2_) and carbon units has been reported in some cancer cells [32]. The conversion of threonine, choline and betaine into glycine has also been reported and may support this process [17]. In our study, in addition to serine, all these initial alternative carbon donors were significantly increased in the IDC samples (*vs*. adjacent tissue), suggesting highly active one-carbon metabolism.

In one-carbon metabolism, serine donates a carbon atom from its side chain to folate, which generates methionine as one of its final products [32]. The folate cycle coupled with the methionine cycle constitutes a bicyclic metabolic pathway that distributes carbon units and methyl groups through a set of metabolic reactions, from the generation of THF and methyl-tetrahydrofolate (mTHF) [19, 32]. The methionine cycle then is linked to the transsulfuration pathway through homocysteine, which can generate glutathione and influence cellular redox regulation [17, 19, 32]. Our metabolomics analysis showed an enrichment in folate, methionine, homocysteine and glutathione metabolism in IDC samples (*vs*. adjacent tissue), suggesting increased activity of all cycles that integrate one-carbon metabolism.

According to the histopathological grade of our samples, more than 70% of the IDC biopsies had a high mitotic index (grades 2 and 3). Proliferating cancer cells generally require nucleotides (purines and pyrimidines) for the synthesis of cellular components, which can involve folate cycle and aspartate metabolism [33–35]. The biosynthesis of polyamines, spermidine and spermine (as well as their precursor, putrescine) and extracellular matrix are also required to ensure the stability and function of nucleotides and to accommodate new tumor cells, which can be supported by the methionine cycle and proline metabolism [36]. In this sense, the increased metabolism of aspartate, proline, purines, as well as increased spermidine and spermine biosynthesis, observed in our IDC biopsies (*vs*. adjacent tissue) were consistent with high cell proliferation rates and might be sustained by one-carbon metabolism activation [32, 33]. This scenario can be validated through gene set enrichment analysis (GSEA) where purine metabolism was highlighted between pathways related to one-carbon metabolism. Interestingly, we observed a decrease in TYMS in our gene validation, which is associated with increased dUMP and DNA instability. This condition results in an imbalance and excessive incorporation of uracil in DNA instead of thymine [37], a process that seems to impair DNA repair mechanisms and contributes to carcinogenesis.

One-carbon metabolism can also directly meet the high metabolite requirements for cell proliferation through its by-product, ammonia [17]. In mice, the ammonia accumulated in the tumor microenvironment is used directly to generate amino acids (recycling) and accelerate the proliferation of breast cancer cells [38]. Accordingly, we identified enrichment of the ammonia recycling in our IDC biopsies along with an increase in the urea cycle (*vs*. adjacent tissue). These findings suggest that the proliferation of IDC cell proliferation implies intense metabolism of amino acids, with a very similar contribution to synthesis and catabolism (protein turnover).

In addition, cancer development and progression require membrane phospholipids, which can also be partially generated by one-carbon metabolism [17]. Comprising approximately 50% of membrane phospholipids, the choline portion of phosphatidylcholines is synthesized through the methionine cycle, while fatty acids are supplied by uptake and/or *de novo* synthesis [17, 39, 40]. Along with the increase in the methionine cycle, we also observed higher levels of saturated fatty acids (SFA) and lower levels of polyunsaturated fatty acids (PUFA) in our IDC biopsies (*vs*. adjacent tissue), which is consistent with previous reports on invasive ductal carcinomas [41]. High SFA levels can contribute to the structure of lipid rafts, membrane microdomains that act as platforms for cell signaling and have been shown to activate oncogenic pathways in breast tumor cells, such as cell proliferation (i.e., from HER-2) [42, 43]. Low PUFA levels may reflect a mechanism for protecting cell membranes from lipid peroxidation [43, 44].

In our study, decreased levels of PUFAs in the IDC included metabolites involved in arachidonic acid (AA, arachidonic acid) metabolism. Enrichment of these PUFAs in the adjacent tissue was previously associated with immune evasion of tumor cells and adjacent tissue inflammation, favoring new mutations [17, 45]. Therefore, our molecular findings suggest a lipid metabolism reprogramming in IDC to serve both cell membrane synthesis and surface protein-mediated signaling for proliferation while favoring cell survival.

Changes in cancer cell metabolism can also influence epigenetic regulation. The methionine cycle activation in IDC (*vs*. adjacent tissue) highlighted by our own analyses provides an important source of substrates for post-translational modifications involving methylation [17, 46]. Arginine methylation is a common post-translational modification that is implicated in different cellular processes that lead to the production of asymmetric dimethyl arginine (ADMA) via nicotinamide adenine dinucleotide phosphate (NADPH) [47, 48]. In our study, a predictive increase in nicotinamide and ADMA metabolism was observed in the IDC samples (*vs*. adjacent tissue) [49]. Increased NADPH availability has been considered an advantage for one-carbon metabolism, since one molecule is consumed in each turn of the cytoplasmic folate cycle [17].

Furthermore, our gene set enrichment analysis identified a predicted activation of the Wnt signaling pathway. A recent study has demonstrated a requirement for methionine cycle activation and ADMA during Wnt signaling, since the depletion of methionine is a potent inhibitor of Wnt signaling and Wnt-induced turnover protein [50].

Increased ADMA production, suggested by our metabolomics data, can generate high levels of ROS [46, 47]. In breast cancers, ROS generation has been associated with cell apoptosis and impaired tumor development [49, 51]. Our IDC biopsies showed a predictive increase in glutathione metabolism (*vs*. adjacent tissue), which is an output of the transsulfuration pathway in one-carbon metabolism and an important regulator of the redox state [20, 47]. This finding suggests an activation of antioxidant defenses due intense cell oxidative stress in IDC to avoid cell apoptosis.

Oxidative stress can lead to mitochondrial damage [52]. Mitochondrial fitness is an important feature of one-carbon metabolism activation, since most of its chemical reactions take place inside these organelles [20, 53]. However, under stress conditions, the one-carbon mitochondrial pathway may be downregulated and its reactions shifted to the cytoplasm [19, 54]. We observed a decrease in the expression of genes that participate in mitochondrial reactions of one-carbon metabolism, such as *SHMT2, GLDC* (glycine dehydrogenase) and *MTHFD2* [19], in parallel to an increase in *ALDH1L1*, which participates in cytoplasmic reactions of one-carbon metabolism by generating tetrahydrofolate (THF) [19]. These findings suggest that major one-carbon activation in IDC takes place in the cytoplasm, probably due oxidative stress.

It is worth noting that our metabolomic data suggest mitochondrial fitness in IDC: activation of the TCA cycle, BCAA degradation, increased succinyl-CoA and α-ketoglutarate metabolites, increased biotin metabolism and enrichment of the malate-aspartate shuttle were observed in this tumor (*vs*. adjacent tissue) [55–58]. These processes may be due to the exclusion of defective mitochondria by autophagy, which is described as a defense mechanism of cancer cells in the presence of ROS accumulation [53]. Taken together, our findings suggest the potential relevance of one-carbon metabolism in IDC cells, allowing carbon unit donation for proliferation processes, even under intense oxidative distress.

Individual analysis of each IDC subtype provided metabolomic observations similar to those obtained in the entire IDC sample. In addition, our metabolomic data showed a strong association of one-carbon activation with cell proliferation, and all studied IDC biopsies were from primary tumors with a marked proliferative status, regardless of their subtype. Considering that high proliferation is a common feature of breast carcinoma development, the set of our metabolomics observations allows the suggestion that activation of the one-carbon pathway is a general property of IDC.

Our study included retrospective data and thus presents the limitations involved in this type of design. However, this enabled us to integrate metabolomic and genomic data from different populations to support the presence of one-carbon activation in IDC. We were also unable to perform a targeted analysis in different breast cancer subtypes, but our untargeted approach strongly suggested that one-carbon activation might be a universal metabolic feature of this malignancy.

Taken together, our results support the ancillary role of several metabolic pathways in breast cancer progression, where one-carbon metabolism activation seems to be pivotal. The extent to which these metabolic pathways represent real vulnerabilities remains to be determined. Considering whether this pathway is sensitive to the availability of nutrients, dietary management should be explored, as well as the effectiveness (from a metabolomic perspective) of antimetabolic drugs, including metformin, 5-FLU, methotrexate, pemetrexed and other agents targeting one-carbon metabolism that have demonstrated clinical benefits [32, 59, 60].

## MATERIALS AND METHODS

### Targeted metabolomics profile

#### Subject selection and sample collection

Between 2007 and 2010, 90 patients with IDC were voluntarily recruited after obtaining written informed consent at the Institut Gustave-Russy and Institut Curie (Paris), University of Debrecen (Hungary), and University of Tartu (Estonia), after ethical approval from the local ethics committee (Reference: CAPPesq 1.560.877). Written informed consent was obtained from each patient prior to trial participation. Invasive ductal carcinoma (IDC) status was confirmed by histopathological analysis. Tissue samples (breast cancer tissue and non-tumor adjacent breast tissue) were collected from the same subjects at the time of diagnosis. The criteria for selection were as follows: at least 35 years old, no macrometastasis disease, no prior anticancer treatment, operable IDC and within stages II and III. Disease diagnosis and staging respectively were performed by histopathological analysis and according to the TNM staging system [61, 62] in size-matched samples of tumor and adjacent (2–5 cm away from the tumor) tissues collected from the same subjects. The IDC (tumor group) and non-tumor adjacent breast tissue (control group) samples were subjected to metabolomics analysis.

#### Targeted metabolomic analyses

Quantitative values of tissue metabolites were obtained from 100 mg of IDC and non-tumor adjacent breast tissue samples by targeted metabolomics analysis. We used different kits customized by BIOCRATES Life Sciences AG, Innsbruck, Austria, which enabled the absolute quantification of >600 different metabolites using mass spectrometry coupled with liquid chromatography (LC) with tandem mass spectrometry (MS/MS) [63, 64].

The sample preparation for analysis was performed according to the kit user manual, which contains an entailed Standard Operating Procedure that was validated by BIOCRATES Life Sciences AG, Innsbruck, Austria, in their labs in Austria. For LC-MS/MS analyses, BIOCRATES (Life Sciences AG, Innsbruck, Austria) kits include a full 7-point calibration curve (run in duplicate; start and end of analysis cohort) and QC samples at low, medium and high concentrations. The QC samples in the kit comprise 57 metabolites that encompass amino acids, biogenic amines, glycerophospholipids and acylcarnitines, with the concentration of the analytes in the three QC samples corresponding to low, medium and high levels relative to the quantitation range for each of these metabolites [11].

The experimental metabolomics measurement technique is described in detail by patent US 2007/0004044 (accessible online at http://www.freepatentsonline.com/20070004044.html). Briefly, a targeted profiling scheme was used to quantitatively screen for fully annotated metabolites using multiple reaction monitoring, neutral loss and precursor ion scans. Quantification of metabolite concentrations and quality control assessment were performed with the MetIQ software package (BIOCRATES Life Sciences AG, Innsbruck, Austria) adhering to 21CFR (Code of Federal Regulations) Part 11, which implies proof of reproducibility within a given error range. Only low-molecular-weight ionizable molecules (m/z <1500) were considered. An xls file was then generated, which contained the sample identification, metabolite names, and tissue metabolite concentrations (expressed in μM).

#### Targeted metabolites panel

The metabolites were quantified using AbsoluteIDQ^®^ p180, which quantified 40 acylcarnitines, 21 amino acids, 19 biogenic amines, the sum of hexoses, 76 phosphatidylcholines, 14 lysophosphatidylcholines, 15 sphingomyelins and 90 glycerophospholipids; AbsoluteIDQ^®^ Stero17, which quantified 17 steroid hormones; Bile Acids kit^®^, which quantified 20 bile acids; the neurotransmitter assay, which quantified 9 neurotransmitters; the eicosanoid assay and other polyunsaturated fatty acid oxidation products (PUFAs), which quantified 17 different types of these molecules; the fatty acid assay, which quantified 32 fatty acids; the lipid assay, which quantified 162 glycerophospholipids, 33 sphingomyelins and 131 ceramides. Glycerophospholipids were differentiated from the presence of ester (a) and ether (e) bonds in the glycerol moiety, where two letters indicate that two glycerol positions were linked to a fatty acid residue (aa = diacyl, ae = acyl-alkyl) and a single letter indicates the presence of a single fatty acid residue (a = acyl or e = alkyl). Additionally, the samples were analyzed for the following metabolites of energy metabolism: lactate, pyruvate/oxaloacetate, α-ketoglutarate, fumarate and succinate (BIOCRATES Life Sciences AG, Innsbruck, Austria) (Supplementary Material 1 – Supplementary Table 4)

#### Targeted metabolomics data analysis

For metabolomics data analysis, only common features found in at least 50% of samples in any group were considered, correcting for individual bias. In the MetaboAnalyst 4.0 (Xia Lab @ McGill, Canada) [65], metabolomic data were previously processed for sample standardization (median normalization), with log-transformation of all quantified metabolites to stabilize the concentration distributions and Pareto Scale, a centered mean and division by the square root of the standard deviation of each variable.

The statistical tools sequentially employed to evaluate significant differences induced by the carcinogenesis process were as follows: PCA (unsupervised multivariate principal component analysis), PLS-DA (supervised multivariate analysis), RF analysis (random forest), and hierarchical analysis, the paired Student’s T and false discovery rate (FDR) - Benjamini-Hochberg test. The most important metabolites, capable of differentiating adjacent normal and tumoral tissues via important variables (VIPs), were selected from the RF and T-test analyses. Complementary analysis for comparison of metabolomic profiles between breast cancer subtypes were also performed using ANOVA and the fold change (FC). The level of statistical significance considered for all tests was 5% (p£0.05). Subsequently, the set of statistically significant metabolites between the groups was submitted to metabolite set enrichment analysis (MSEA), which was also performed using the MetaboAnalyst 4.0 platform.

#### Gene validation

Gene set enrichment analysis was performed by GSEA (Broad Institute) using the KEGG and REACTOME databases. TCGA-BRCA data were downloaded by TCGAbiolinks. DESeq2 was used to perform gene differential expression [25].

### Untargeted metabolomics profile

#### Subject selection and sample collection

Forty-two samples of paraffin-block frozen breast cancer subtypes were selected from the AC Camargo Cancer Canter biobank after ethical approval from the local ethics committee (Reference: Project Alias WSSS1944 - code 2165/16). The tumor tissue samples were classified as luminal subtype A (*n =* 11), luminal B (*n =* 11), triple negative (*n =* 10) and HER2 positive (*n =* 10). All samples belonged to female patients with a primary tumor, infiltrative ductal, without distant metastasis and without previous treatment status. Written informed consent was obtained from each patient prior to trial participation.

#### Sample preparation for untargeted metabolomic analyses

Tumor samples were obtained from the Tissue-Tek OCT matrix and washed five times with 200 μL of PBS buffer (NaCl 0.137 mol/L, KCl 0.0027 mol/L, Na2HPO4, 0.01, KH2PO4 0.0018 mol/L, pH ?7.4). Then, they were dried with an Eppendorf Concentrator plus (Hamburg, Germany) and weighed. Metabolite extraction was perform by adding 10 μL of MeOH: CHCl_3_: H_2_O 6:3:1 (v/v) per mg of dried tissue in a Bullet Blender (Next, Advance, Troy, NY, USA) with stainless steel beads using five cycles of 5 minutes (maximum speed). The supernatant was dried and resuspended in 20 μL of 1:1 water: acetonitrile (v/v) containing 150 μmol/L internal standard (p-fluoro-phenylalanine) per mg of dried tissue. The solution was centrifuged (15,000 rpm, 10 minutes, 4 °C), and the supernatant was analyzed.

#### Untargeted metabolomic analyses by LC-MS

LC-MS analysis was performed using high-performance liquid chromatography (Prominence, Shimadzu, Kyoto, Japan) coupled to a quadrupole-time-of-flight mass spectrometer (microQTOF II, Bruker Daltonics, Bremen, Germany) using electrospray ionization. A 3-μL sample volume was injected into a Kinetex C18 column (Phenomenex, Torrance, CA, EUA, 2.1 × 150 mm, 2.6 μm) at 40° C using mobile phase A (0.1% formic acid aqueous solution, v/v) and mobile phase B (0.1% formic acid in acetonitrile v/v) at a flow rate of 0.2 mL/min with the following gradient: 0–3 min (5–20% B), 3–10 min (20–100% B), 10–15 (100% B), 15.–15.1 (100–5% B), 15.1–23 min (5% B). MS analysis was carried out in positive and negative mode in full scan mode from m/z 70 to 1000 at a spectrum rate of 2 Hz. The end plate offset was 500 V, hexapole RF was 120 Vpp (positive mode) and 200 Vpp (negative mode), and capillary was 4500 V (positive) and 3500 V (negative). For ESI, a gas temperature of 250° C, gas flow of 8 L/min and pressure of 4 bar were applied.

#### Untargeted metabolomics data treatment

Data processing was performed using the XCMS package [66] on the R platform (3.2.3, R Foundation for Statistical Computing, R Core Team). LC-MS data were converted to a *. mzXML file using Bruker Compass DataAnalysis 4.0 software (Bruker Daltonics, Germany).

For both positive and ionization modes, molecular features (MF) were extracted by applying the matched filter algorithm in the XCMS software using a signal/noise threshold (snthresh = 10), full width at half maximum of the model peak (fwhm = 4), and minimum difference in m/z for peaks with overlapping retention times (mzdiff = 0.01), between 1 and 18 minutes. For the grouping step, the band width (bw = 20 and 10, for the first and second grouping, respectively) and width of overlapping m/z slices (mzwid = 0.025) were used. The data were aligned using the retcor algorithm with a smooth loess and 0.5 span. After the second grouping, fill Peaks was used to reduce missing values. Raw data were normalized to the intensity of the internal standard, fluoro-phenylalanine (m/z [M+H] + =184.0768 and [M-H]- = 182.0623, for positive and negative data, respectively).

#### Untargeted statistical analysis

Principal component analysis (PCA) and partial least squares discriminant analysis (PLS-DA) were carried out after log transformation and Pareto scaling to evaluate group separations in SIMCA P+ (version 12.0.1, Umetrics, Sweden). Significant statistical MFs were selected according to the variable importance projection (VIP score > 1.0) observed in the PLS-DA models.

Differences for individual MFs were evaluated by comparing groups using univariate analysis, in which the Mann-Whitney U test (*p*-value < 0.05) Statistica 10 software and the t test (*p*-value < 0.05) were applied.

#### Untargeted metabolite annotation

Statistically significant MF, both positive and negative modes, was putatively identified in the CEU mass mediator, version 3.0 [67] by searching the Human Metabolome Database (HMDB) and Metlin databases. The metabolites were annotated using [M+H]+, [M+2H]2+, and [M+Na]+ as possible adducts for positive mode and [M–H]–, [M+FA–H]–, and [M–2H]2– for negative mode. The tolerance error used was 5 ppm. The putatively identified metabolites were correlated with metabolic pathways using the Kyoto Encyclopedia of Genes and Genome, KEGG (https://www.genome.jp/kegg/) database.

## SUPPLEMENTARY MATERIALS








