# Supplementary material for: Distinct pattern of one-carbon metabolism, a nutrient-sensitive pathway, in invasive breast cancer: A metabolomic study
Source: Oncotarget. 2020 May 5;11(18):1637–52. doi: 10.18632/oncotarget.27575 (PMC7210010; doi:10.18632/oncotarget.27575)
Supplement: Supplementary file 1 [file oncotarget-11-1637-s001.pdf]

Distinct pattern of one-carbon metabolism, a nutrient-sensitive pathway, in invasive breast cancer: A metabolomic study

SUPPLEMENTARY MATERIALS

SUPPLEMENTARY MATERIAL 1

PLS-DA cross validation details:

Measure	1 comps	2 comps	3 comps	4 comps	5 comps
Accuracy	0.94667	0.96333	0.96667	0.99	0.98667
R2	0.76411	0.82589	0.87762	0.91483	0.93106
Q2	0.72939	0.77135	0.80968	0.82294	0.82489

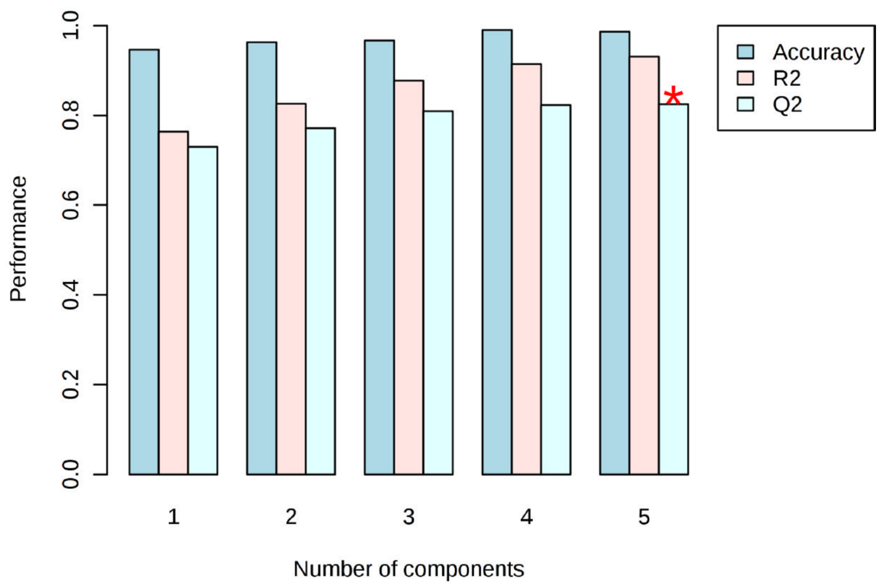

Supplementary Figure 1: Q2 is an estimate of the predictive ability of the model and is calculated via cross-validation (CV). In each CV, the predicted data are compared with the original data, and the sum of squared errors is calculated. The prediction error is then summed over all samples (Predicted Residual Sum of Squares or PRESS). For convenience, the PRESS is divided by the initial sum of squares and subtracted from 1 to resemble the scale of the R2. Good predictions have high Q2, as noted in our figure.

Supplementary Table 1: Tissue metabolites differentiating breast cancer tissue from non-tumor adjacent breast tissue. Differential 99 metabolites identified using LC-MS/MS. See Supplementary Table 1

Supplementary Table 2: Detailed Metabolite Set Enrichment Analysis (MSEA) including all the 55 identified pathways in IDC

#	Pathway	Hits	Expect	*P* value	FDR
1	Ammonia Recycling	32	8	2.06	0.058
2	Urea Cycle	29	7	1.87	0.163
3	Aspartate Metabolism	35	7	2.26	0.51
4	Glycine and Serine Metabolism	59	9	3.8	0.987
5	Carnitine Synthesis	22	5	1.42	1.0
6	Arginine and Proline Metabolism	53	8	3.42	1.0
7	Alanine Metabolism	17	4	1.1	1.0
8	Oxidation of Branched Chain Fatty Acids	26	5	1.68	1.0
9	Malate-Aspartate Shuttle	10	3	0.645	1.0
10	Spermidine and Spermine Biosynthesis	18	4	1.16	1.0
11	Glutamate Metabolism	49	7	3.16	1.0
12	Glucose-Alanine Cycle	13	3	0.838	1.0
13	Methionine Metabolism	43	6	2.77	1.0
14	Phenylalanine and Tyrosine Metabolism	28	4	1.8	1.0
15	Glutathione Metabolism	21	3	1.35	1.0
16	Beta-Alanine Metabolism	34	4	2.19	1.0
17	Valine, Leucine and Isoleucine Degradation	60	6	3.87	1.0
18	Methylhistidine Metabolism	4	1	0.258	1.0
19	Beta Oxidation of Very Long Chain Fatty Acids	17	2	1.1	1.0
20	Lysine Degradation	30	3	1.93	1.0
21	Warburg Effect	58	5	3.74	1.0
22	Tryptophan Metabolism	60	5	3.87	1.0
23	Biotin Metabolism	8	1	0.516	1.0
24	Homocysteine Degradation	9	1	0.58	1.0
25	Phenylacetate Metabolism	9	1	0.58	1.0
26	Cysteine Metabolism	26	2	1.68	1.0
27	Phytanic Acid Peroxisomal Oxidation	26	2	1.68	1.0
28	Propanoate Metabolism	42	3	2.71	1.0
29	Mitochondrial Beta-Oxidation of Long Chain Saturated Fatty Acids	28	2	1.8	1.0
30	Selenoamino Acid Metabolism	28	2	1.8	1.0
31	Phosphatidylethanolamine Biosynthesis * endocanabionoides - imunossupressão	12	1	0.773	1.0
32	Taurine and Hypotaurine Metabolism	12	1	0.773	1.0
33	Ketone Body Metabolism	13	1	0.838	1.0
34	Thyroid hormone synthesis	13	1	0.838	1.0
35	Citric Acid Cycle	32	2	2.06	1.0
36	Amino Sugar Metabolism	33	2	2.13	1.0
37	Gluconeogenesis	35	2	2.26	1.0
38	Tyrosine Metabolism	72	4	4.64	1.0
39	Nicotinate and Nicotinamide Metabolism	37	2	2.38	1.0
40	Purine Metabolism	74	4	4.77	1.0
41	Butyrate Metabolism	19	1	1.22	1.0
42	Mitochondrial Electron Transport Chain	19	1	1.22	1.0
43	Catecholamine Biosynthesis	20	1	1.29	1.0
44	Threonine and 2-Oxobutanoate Degradation	20	1	1.29	1.0
45	Betaine Metabolism	21	1	1.35	1.0
46	Fatty acid Metabolism	43	2	2.77	1.0
47	Histidine Metabolism	43	2	2.77	1.0
48	Mitochondrial Beta-Oxidation of Short Chain Saturated Fatty Acids	27	1	1.74	1.0
49	Folate Metabolism	29	1	1.87	1.0
50	Porphyrin Metabolism	40	1	2.58	1.0
51	Sphingolipid Metabolism	40	1	2.58	1.0
52	Bile Acid Biosynthesis	65	2	4.19	1.0
53	Pyruvate Metabolism	48	1	3.09	1.0
54	Pyrimidine Metabolism	59	1	3.8	1.0
55	Arachidonic Acid Metabolism	69	1	4.45	1.0

Legend: HITS: actually matched number from the data; Expected: number of metabolites expected for metabolic pathway; P value: p values obtained after performing t-test (p-value<0.05); FDR: value obtained after performing false discovery test.

Supplementary Table 3: Clinical pathological characteristics IDC patients

	BREAST CANCER PACIENTS (N = 90)
AGE	55 (mediam)
Menopause status	
Peri	4
yes	51
no	35
FAMILY HISTORY	
yes	28
no	62
subtype	
LUMINAL B-HER2	10
LUMINAL A	47
LUMINAL B	5
HER2	8
NEGATIVE TRIPLE	20
tumor size (MM)	
5-19	31
20-49	48
50+	6
date not collected	5
GRADE	
I	0
II	22
III	68
MITOTIC INDEX	
1	18
2	20
3	52
cOMMON LYMPHONOD	
pN0	33
pN1	43
pN2	14
DATE NOT COLLECTED	2

Supplementary Table 4: Biocrates' targeted metabolomics approach kits immediate identification of more than 630 endogenous metabolites of different classes, measurement of their absolute concentrations. See Supplementary Table 4

SUPPLEMENTARY MATERIAL 2

See Supplementary Material 2

SUPPLEMENTARY MATERIAL 3

Principal component analysis (PCA) and Partial Least Squares Discriminant Analysis (PLS-DA), heatmap, and differentially expressed metabolites were performed after Pareto normalization to the raw data. Student's T-test was applied when comparing between surrounding (*n* = 42) and tumor (*n* = 47). Adjusted p values were obtained applying the false discovery rate (FDR) method (Benjamini & Hochberg). The data was considered significative when FDR <= 0.05 and log2 FC >= 2. R v3.5.1 was used to run analysis; graphics were generated by the following R packages: ggplot2, and "heatmap".

REFERENCES

1. Grace SC, Hudson DA. Processing and visualization of metabolomics data using R. Fundamentals and Applications. 2016. https://doi.org/10.5772/65405.

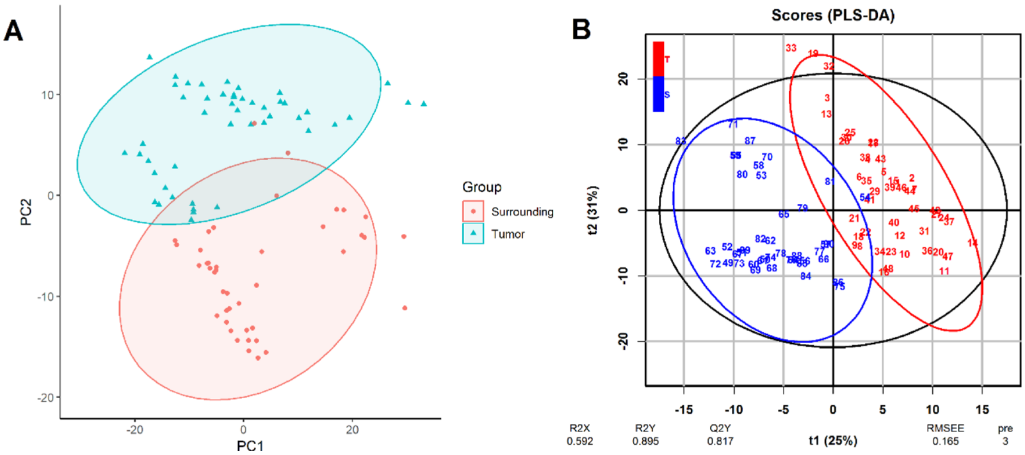

Supplementary Figure 1: Distinct one-carbon metabolites from surrounding normal and tumor. (**A**) Principal component analysis (PCA) from metabolites obtained from the surrounding (red) and tumor (blue) samples. (**B**) Partial Least Squares Discriminant Analysis (PLS-DA) identifies the tumor (tumor) and surrounding normal tissue (red).

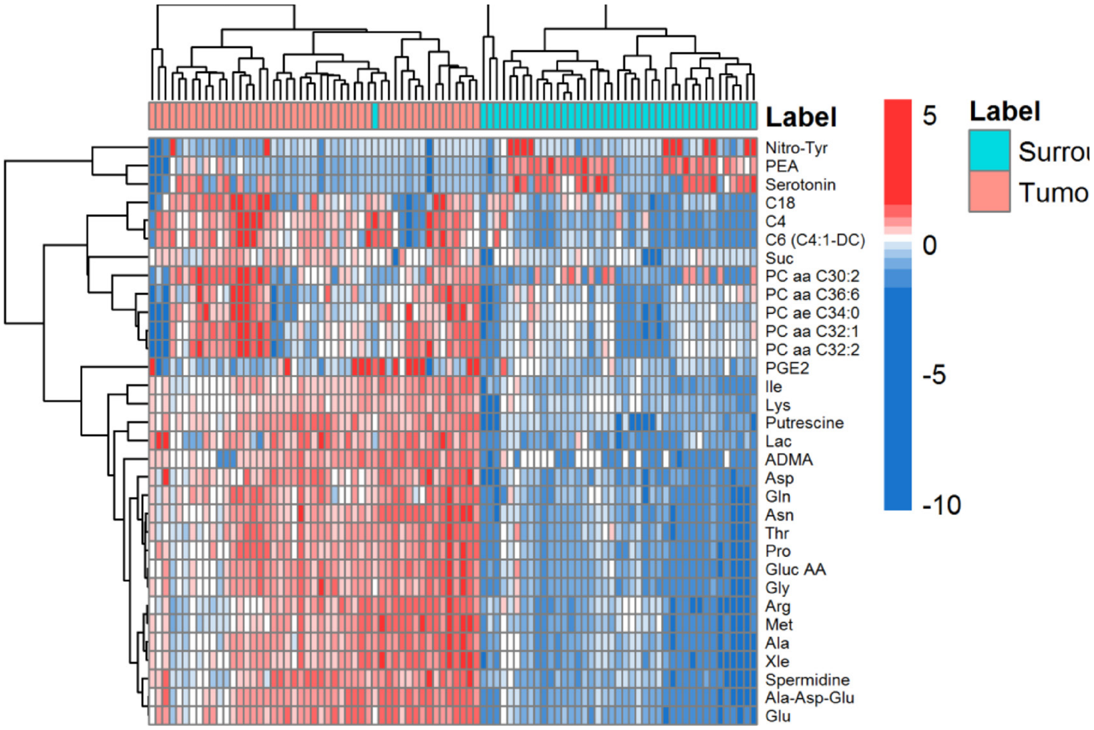

Supplementary Figure 2: Differentially expressed metabolites between surrounding tissue and tumor from breast cancer patients. Normal adjacent tumor metabolites are very different from tumor metabolites. Dendrograms were build based on Pearson correlation test. Only top differentially expressed metabolites are depicted (FDR < 0.05, log2FoldChange >2).
