# Supplementary material for: Distinct pattern of one-carbon metabolism, a nutrient-sensitive pathway, in invasive breast cancer: A metabolomic study
Source: Oncotarget. 2020 May 5;11(18):1637–52. doi: 10.18632/oncotarget.27575 (PMC7210010; doi:10.18632/oncotarget.27575)
Supplement: Supplementary file 2 [file oncotarget-11-1637-s002.docx]

**Supplementary Table 1:** Tissue metabolites differentiating breast cancer tissue from non-tumor adjacent breast tissue. Differential 99 metabolites identified using LC-MS/MS.

| Metabolite | Name | HMDM ID | *P Value* | FDR | FC | Reg.  BC-T |
| --- | --- | --- | --- | --- | --- | --- |
| CARNITINAS | | | | | | |
| C0 | L-Carnitine | HMDB0000062 | 3.3214e-06 | 1.5074E-5 | -- | ↑ |
| C10 | Decanoylcarnitine | HMDB0000651 | 0.00025834 | 8.5471E-4 | -- | ↓ |
| C10:1 | Decenoylcarnitine | HMDB0013205 | 3.4638e-06 | 1.5521E-5 | -- | ↓ |
| C12:1 | **Dodecanoylcarnitine** | HMDB0002250 | 0.0048805 | 0.012082 | -- | ↓ |
| C14:2 | Tetradecadienylcarnitine | HMDB0013331 | 2.8442e-05 | 1.0826E-4 | -- | ↓ |
| C14:2-OH | Hydroxytetradecadienylcarnitine | HMDB0062588 | 0.0022748 | 0.0060096 | 0.27319 | ↓ |
| C16 | Palmitoylcarnitine | HMDB0000222 | 0.0058466 | 0.014176 | 21.757 | ↑ |
| C18 | Stearoylcarnitine | HMDB0000848 | 0.0004725 | 0.0014547 | 31.094 | ↑ |
| C2 | Acetylcarnitine | HMDB0000201 | 9.8737e-06 | 4.0176E-5 | 23.706 | ↑ |
| C3 | Propionylcarnitine | HMDB0000824 | 0.0019687 | 0.0052524 | -- | ↑ |
| C3-OH | Hydroxypropionylcarnitine | HMDB0013125 | 0.019476 | 0.041038 | -- | ↓ |
| C4 | Butyrylcarnitine | HMDB0002013 | 1.0438e-10 | 9.0126E-10 | 35.033 | ↑ |
| C5-DC (C6-OH) | Glutarylcarnitine | HMDB0013130 | 6.1354e-06 | 2.6487E-5 | -- | ↑ |
| C5-M-DC | Methylglutarylcarnitine | HMDB0000552 | 0.0093616 | 0.02152 | -- | ↓ |
| C5-OH (C3-DC-M) | Hydroxyvalerylcarnitine | HMDB0013132 | 8.8963e-06 | 3.705E-5 | 22.588 | ↑ |
| C6 (C4:1-DC) | Caproylcarnitine (**Hexanoylcarnitine)** | HMDB0000705 | 1.5583e-08 | 9.8504E-8 | -- | ↑ |
| *C9* | Nonaylcarnitine | HMDB0013288 | 0.0018522 | 0.0050775 | -- | ↓ |
| AA e AB | | | | | |  |
| ADMA | Asymmetric dimethylarginine | HMDB0001539 | 5.806e-13 | 7.3405E-12 | 19.469 | ↑ |
| Ala | Alanine | HMDB0000161 | 3.1672e-15 | 7.0075E-14 | 41.563 | ↑ |
| Arg | Arginine | HMDB0000517 | 8.5278e-10 | 6.5627E-9 | 31.418 | ↑ |
| Asn | Asparagine | HMDB0000168 | 1.485e-20 | 5.2568E-19 | 46.432 | ↑ |
| Asp | **Aspartate** | HMDB0000191 | 4.8124e-22 | 2.4337E-20 | 12.524 | ↑ |
| alpha-KGA | Oxoglutaric acid | HMDB0000208 | 0.017554 | 0.038123 | 12.359 | ↑ |
| Cit | Citrulline | HMDB0000904 | 0.013627 | 0.030151 | -- | ↑ |
| Gln | Glutamine | HMDB0000641 | 2.0831e-18 | 5.6723E-17 | 38.825 | ↑ |
| Glu | Glutamate | HMDB0000148 | 6.0883e-24 | 5.3881E-22 | 99.616 | ↑ |
| Gly | Glycine | HMDB0000123 | 1.5345e-22 | 1.0864E-20 | 78.578 | ↑ |
| His | Histidine | HMDB0000177 | 3.3125e-10 | 2.727E-9 | 23.631 | ↑ |
| Kyn | Kynurenine | HMDB0000684 | 0.01887 | 0.040403 | --- | ↑ |
| Lac | Lactate (Lactic acid) | HMDB0000190 | 2.6137e-16 | 6.609E-15 | 18.465 | ↑ |
| Leu | Leucine | HMDB0000687 | 1.4903e-13 | 2.5123E-12 | 30.532 | ↑ |
| Ile | Isoleucine | HMDB0000172 | 2.0693e-06 | 9.7673E-6 | 39.912 | ↑ |
| Lys | Lysine | HMDB0000182 | 1.5795e-05 | 6.0774E-5 | 43.761 | ↑ |
| Met | Methionine | HMDB0000696 | 1.3633e-11 | 1.3406E-10 | 39.028 | ↑ |
| Nitro-Tyr | Nitrotyrosine | HMDB0001904 | 0.00026981 | 8.6829E-4 | 0.070651 | ↓ |
| PEA | Phenylethylamine | HMDB0012275 | 1.5117e-07 | 8.3616E-7 | 0.085832 | ↓ |
| Phe | Phenylalanine | HMDB0000159 | 9.7074e-13 | 1.185E-11 | 29.721 | ↑ |
| Pro | Proline | HMDB0000162 | 6.2455e-20 | 2.0099E-18 | 46.891 | ↑ |
| Putrescine | Putrescine | HMDB0001414 | 7.5259e-12 | 7.8358E-11 | 3.351 | ↑ |
| Sarcosine | Sarcosinate | HMDB0000271 | 0.00026348 | 8.6364E-4 | -- | ↑ |
| Ser | Serine | HMDB0000187 | 2.7281e-11 | 2.5414E-10 | 29.809 | ↑ |
| Serotonin | Serotonin | HMDB0000259 | 7.8817e-06 | 3.3216E-5 | 0.12851 | ↓ |
| Spermidine | Spermidine | HMDB0001257 | 3.2053e-13 | 4.3641E-12 | 54.818 | ↑ |
| Spermine | Spermine | HMDB0001256 | 6.8351e-08 | 4.0327E-7 | 45.088 | ↑ |
| Taurine | Taurine | HMDB0000251 | 0.0001114 | 3.9046E-4 | -- | ↑ |
| Thr | Threonine | HMDB0000167 | 3.7902e-16 | 8.9449E-15 | 38.468 | ↑ |
| Trp | Tryptophan | HMDB0000929 | 2.2204e-07 | 1.2093E-6 | -- | ↑ |
| Tyr | Tyrosine | HMDB0000158 | 4.3121e-13 | 5.6537E-12 | 2.936 | ↑ |
| Val | Valine | HMDB0000883 | 9.7074e-13 | 6.5574E-11 | 2.981 | ↑ |
| Suc | Succinic acid | HMDB0000254 | 0.0032997 | 0.0084036 | 36.703 | ↑ |
| 13s-HODE | **13S-hydroxyoctadecadienoic acid** | HMDB0004667 | 7.0576E-4 | 0.0021354 | -- | ↓ |
| **HEXOSE** | | | | | | |
| GCKR | Soma de Hexoses  (Incluindo Glicose) | - | 2.5333e-13 | 3.5871E-12 | 0.31975 | ↓ |
| SPHINGOLIPIDS | | | | | | |
| SM C18:1 | **SM(d18:1/18:1(9Z))** | HMDB0012101 | 4.4021e-10 | 3.5417E-9 | 0.38281 | ↓ |
| SM C16:1 | **SM(d18:0/16:1(9Z))** | HMDB0013464 | 9.2127e-10 | 6.9389E-9 | 0.41946 | ↓ |
| SM C22:1-OH | **SM(d18:0/24:1(15Z)(OH))** | HMDB0013469 | 5.304e-06 | 2.3181E-5 | 0.4944 | ↓ |
| SM C22:2-OH | SM(d18:1/24:1(15Z)) | HMDB0012107 | 2.5352e-09 | 1.8697E-8 | 0.4205 | ↓ |
| SM C18:0 | **SM(d18:0/18:0)** | HMDB0012087 | 0.0016394 | 0.0045697 | -- | ↓ |
| SM C20:2 | SM(D18:0/20:2(11Z,14Z)) | HMDB0013465 | 0.0044364 | 0.01106 | -- | ↓ |
| PHOSPHOLIPIDS | | | | | | |
| lysoPC a C18:2 | LysoPC(18:2(9Z,12Z)) | HMDB0010386 | 1.2771e-07 | 7.1758E-7 | -- | ↓ |
| lysoPC a C20:4 | LysoPC(20:4(5Z,8Z,11Z,14Z)) | HMDB0010395 | 4.9868e-06 | 2.2066E-5 | 0.49667 | ↓ |
| lysoPC a C28:0 | lysoPC(28:0) | HMDB0029206 | 0.00031483 | 9.951E-4 | -- | ↑ |
| lysoPC a C14:0 | **LysoPC(14:0/0:0)** | HMDB0010379 | 0.00073737 | 0.0022121 | -- | ↓ |
| lysoPC a C28:1 | LysoPC a C28:1 | HMDB0029221 | 0.0018789 | 0.0050775 | -- | ↑ |
| lysoPC a C16:1 | LPC(16:1) | HMDB0010383 | 0.0035229 | 0.0089078 | -- | ↑ |
| PC ae C34:3 | **PC(o-16:1(9Z)/18:2(9Z,12Z))** | HMDB0013413 | 1.4263e-20 | 5.2568E-19 | 0.17503 | ↓ |
| PC ae C42:1 | PC(18:1(9Z)/24:0) | HMDB0008124 | 1.5247e-11 | 1.4587E-10 | 23.219 | ↑ |
| PC ae C36:0 | Phosphatidylcholine(36:0) | HMDB0007886 | 4.3633e-09 | 3.0892E-8 | 26.305 | ↑ |
| PC ae C30:0 | Phosphatidylcholine(30:0) | HMDB0007869 | 2.9032e-07 | 1.5572E-6 | 21.835 | ↑ |
| PC aa C32:1 | PC(14:0/18:1(9Z)) | HMDB0007873 | 1.0255e-06 | 5.1863E-6 | 2.853 | ↑ |
| PC ae C36:5 | **PC(o-16:1(9Z)/20:4(8Z,11Z,14Z,17Z))** | HMDB0013415 | 1.5229e-06 | 7.4877E-6 | 0.47189 | ↓ |
| PC aa C40:3 | Phosphatidylcholine(40:3) | HMDB0008086 | 2.494e-06 | 1.1466E-5 | 21.341 | ↑ |
| PC aa C30:2 | PC(16:1(9Z)/14:1(9Z)) | HMDB0007999 | 9.2648e-06 | 3.8137E-5 | 42.182 | ↑ |
| PC ae C36:3 | **PC(o-18:1(9Z)/18:2(9Z,12Z))** | HMDB0013429 | 1.1172e-05 | 4.479E-5 | -- | ↓ |
| PC ae C34:0 | Phosphatidylcholine(34:0) | HMDB0007878 | 1.3126e-05 | 5.163E-5 | 2.713 | ↑ |
| PC aa C40:4 | PC(20:4(5Z,8Z,11Z,14Z)/20:0) | HMDB0008438 | 1.5074e-05 | 5.864E-5 | -- | ↑ |
| PC aa C32:2 | PC(14:0/18:2(9Z,12Z)) | HMDB0007874 | 3.3458e-05 | 1.2468E-4 | 27.925 | ↑ |
| PC aa C34:1 | PC(16:1(9Z)/P-18:0) | HMDB0008028 | 0.00016654 | 5.6687E-4 | -- | ↑ |
| PC ae C38:6 | 1-Palmityl-2-docosahexaenoyl-sn-glycero-3-phosphocholine | HMDB0013409 | 0.00020708 | 6.9817E-4 | -- | ↓ |
| PC aa C40:2 | PC(18:2(9Z,12Z)/22:0) | HMDB0008150 | 0.00027566 | 8.7914E-4 | 2.051 | ↑ |
| PC ae C34:1 | PC(o-16:1(9Z)/18:0) | HMDB0013412 | 0.00044131 | 0.0013704 | 20.298 | ↑ |
| PC aa C36:6 | PC(18:2(9Z,12Z)/18:4(6Z,9Z,12Z,15Z)) | HMDB0008142 | 0.00058899 | 0.0017974 | 31.515 | ↑ |
| PC aa C38:1 | PC(18:0/20:1(11Z)) | HMDB0008044 | 0.00079705 | 0.002371 | -- | ↑ |
| PC aa C24:0 | 1-tetradecanoyl-2-decanoyl-sn-glycero-3-phosphocholine | * | 0.0008608 | 0.0025394 | -- | ↓ |
| PC ae C38:0 | PC(o-18:0/20:0) | HMDB0013419 | 0.0011155 | 0.0032106 | -- | ↑ |
| PC aa C42:4 | PC(22:1(13Z)/20:3(5Z,8Z,11Z)) | HMDB0008572 | 0.0011397 | 0.0032536 | -- | ↑ |
| PC ae C36:1 | PC(o-18:1(9Z)/18:0) | HMDB0013427 | 0.0015065 | 0.0042325 | -- | ↑ |
| PC aa C28:1 | PC(14:1(9Z)/14:0) | HMDB0007899 | 0.0018148 | 0.0050189 | -- | ↓ |
| PC ae C34:2 | PC(O-16:0/18:2(9Z,12Z)) | HMDB0011151 | 0.0030729 | 0.0079402 | -- | ↓ |
| PC aa C26:0 | 1-tetradecanoyl-2-dodecanoyl-sn-glycero-3-phosphocholine | * | 0.0055853 | 0.013672 | -- | ↓ |
| PC aa C34:4 | PC(20:3(5Z,8Z,11Z)/14:1(9Z)) | HMDB0008362 | 0.0097275 | 0.022216 | -- | ↑ |
| PC ae C42:2 | PC(o-18:2(9Z,12Z)/24:0) | HMDB0013438 | 0.011911 | 0.026858 | -- | ↑ |
| PC ae C38:2 | PC(o-18:1(9Z)/20:1(11Z)) | HMDB0013431 | 0.012539 | 0.028094 | -- | ↑ |
| PC aa C36:5 | PC(P-18:1(11Z)/18:4(6Z,9Z,12Z,15Z)) | HMDB0011280 | 0.014215 | 0.031255 | -- | ↓ |
| PC aa C38:3 | PC(18:1(9Z)/20:2(11Z,14Z)) | HMDB0008111 | 0.01521 | 0.033236 | -- | ↑ |
| PC aa C30:0 | **PC(P-16:0/14:0)** | HMDB0011203 | 0.020546 | 0.042724 | -- | ↑ |
| PC ae C36:4 | PC(o-18:2(9Z,12Z)/18:2(9Z,12Z)) | HMDB0013435 | 0.020638 | 0.042724 | -- | ↓ |
| PC aa C36:1 | PC(14:0/22:1(13Z)) | HMDB0007887 | 0.022924 | 0.046908 | -- | ↑ |
| PC ae C42:0 | PC(o-18:0/24:0) | HMDB0013423 | 0.023853 | 0.048528 | -- | ↑ |

**Legend***:* HMDB ID: Metabolite ID obtained from HMDB database; p value: p values obtained after performing t-test (p-value<0.05); FDR: value obtained after performing false discovery test; FC (Fold change) measure describing the degree of quantity change between BC-T and BS-S.: Reg. (BC-T vs BS-S): Regulation between breast cancer tissue and non-tumor adjacent breast tissue. * Date not found.

Briefly, the lipid side chain composition was denoted “Cx:y” where x and y refer to the number of carbon atoms and double bonds, respectively. Acylcarnitines, glycerophospholipids and sphingolipids were abbreviated according to the fatty acid side chain. All glycerophospholipids in the panel were phosphatidylcholines. Phosphatidylcholines with one fatty acid side chain bound with an acyl bond are labelled as “LysoPC”, “PC aa” indicates 2 acyl side chains, and “PC ae” indicates one acyl and one alkyl side chain. Amino acids were labelled with standard abbreviations.
