# Supplementary material for: Distinct pattern of one-carbon metabolism, a nutrient-sensitive pathway, in invasive breast cancer: A metabolomic study
Source: Oncotarget. 2020 May 5;11(18):1637–52. doi: 10.18632/oncotarget.27575 (PMC7210010; doi:10.18632/oncotarget.27575)
Supplement: Supplementary file 3 [file oncotarget-11-1637-s003.docx]

**Supplementary Table 4:** Biocrates' targeted metabolomics approach kits immediate identification of more than 630 endogenous metabolites of different classes, measurement of their absolute concentrations. The following is a description of each kit used:


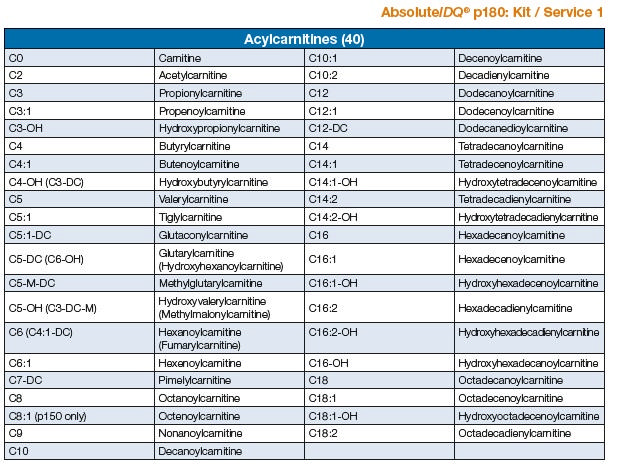


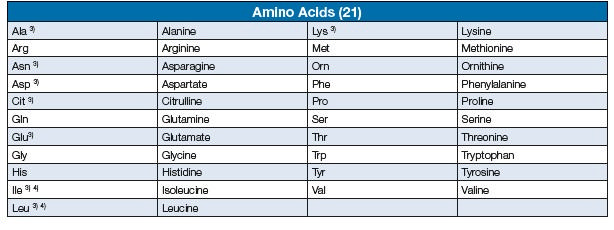


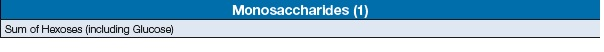


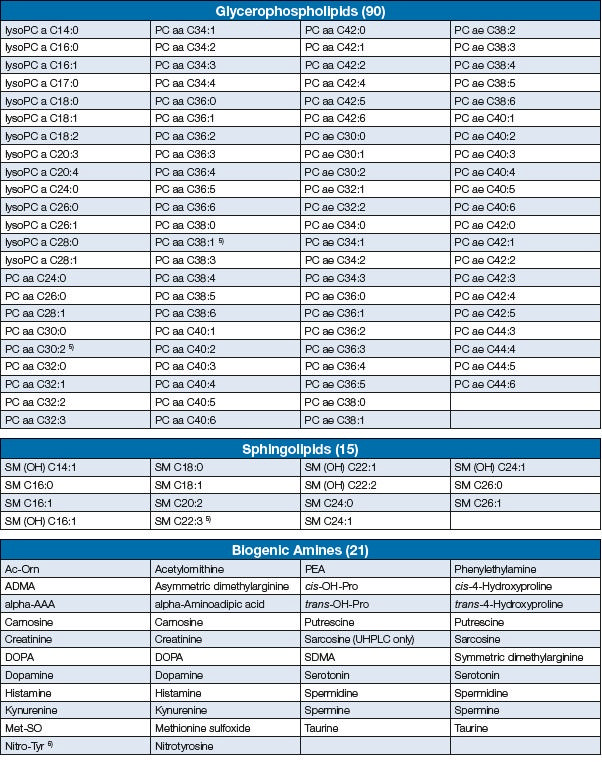


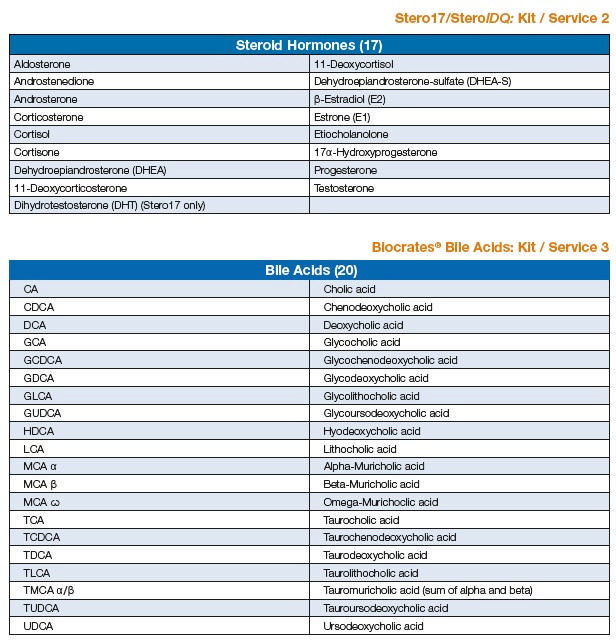


**
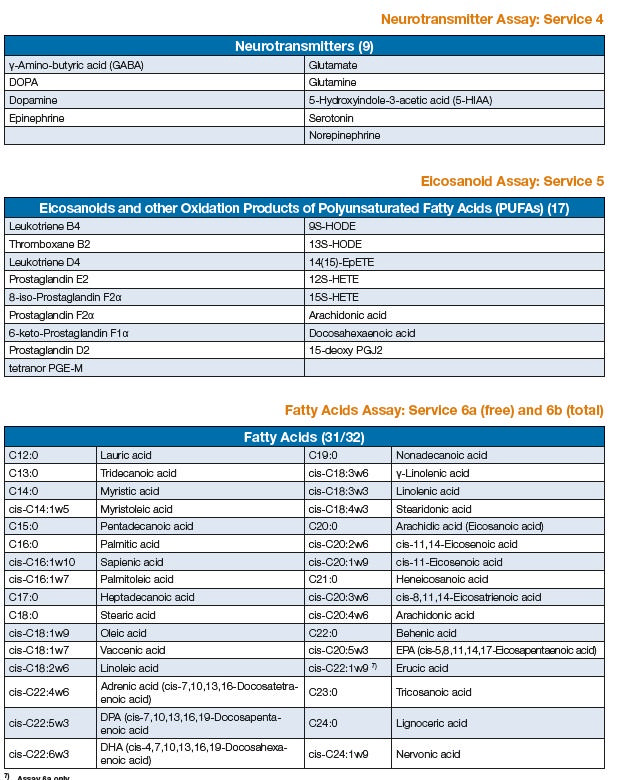
**

**
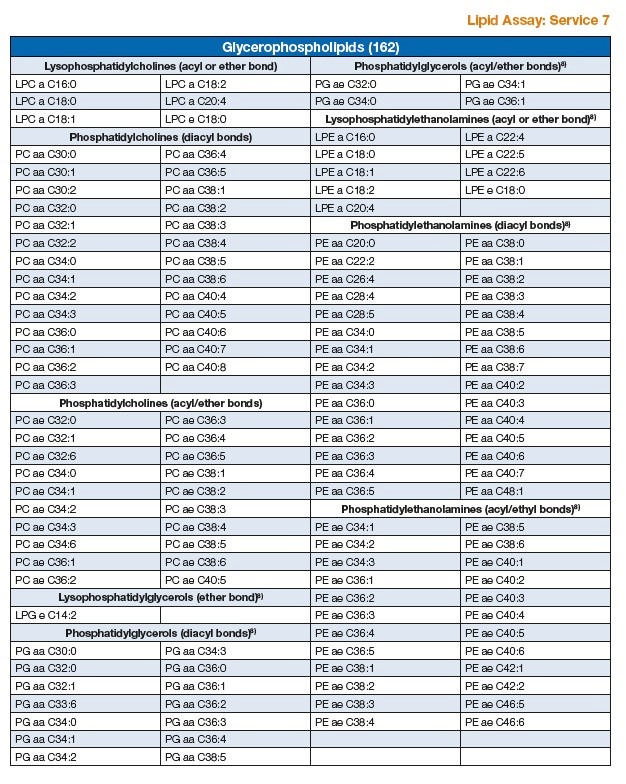
**

**
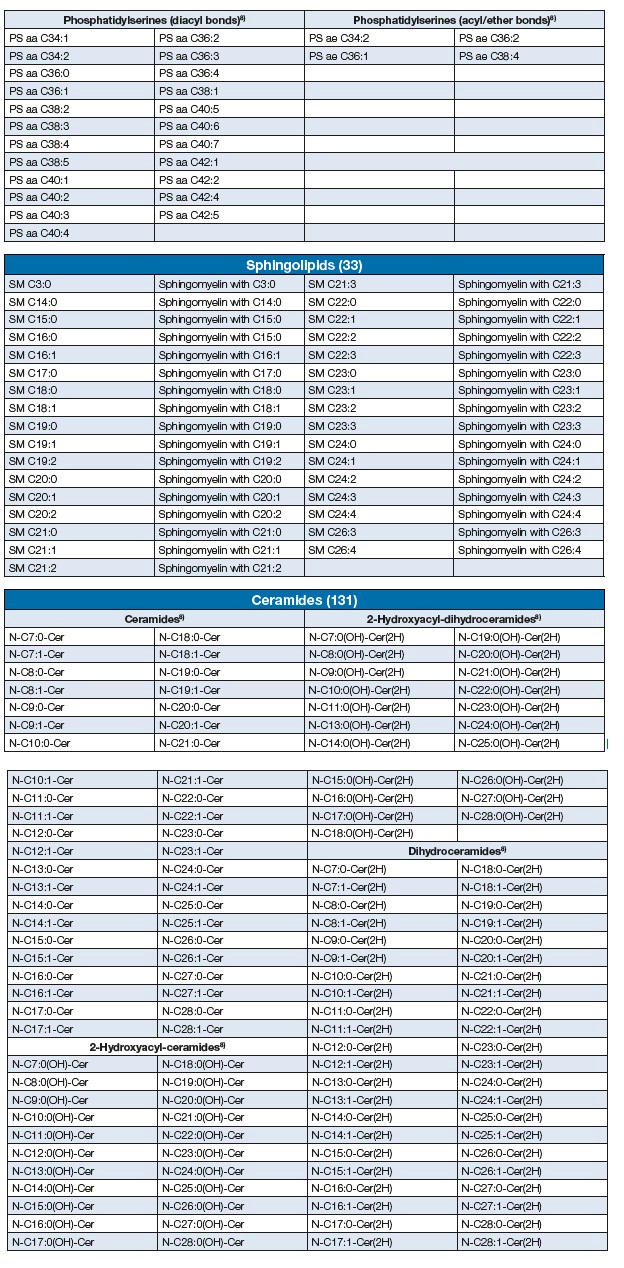
**

**
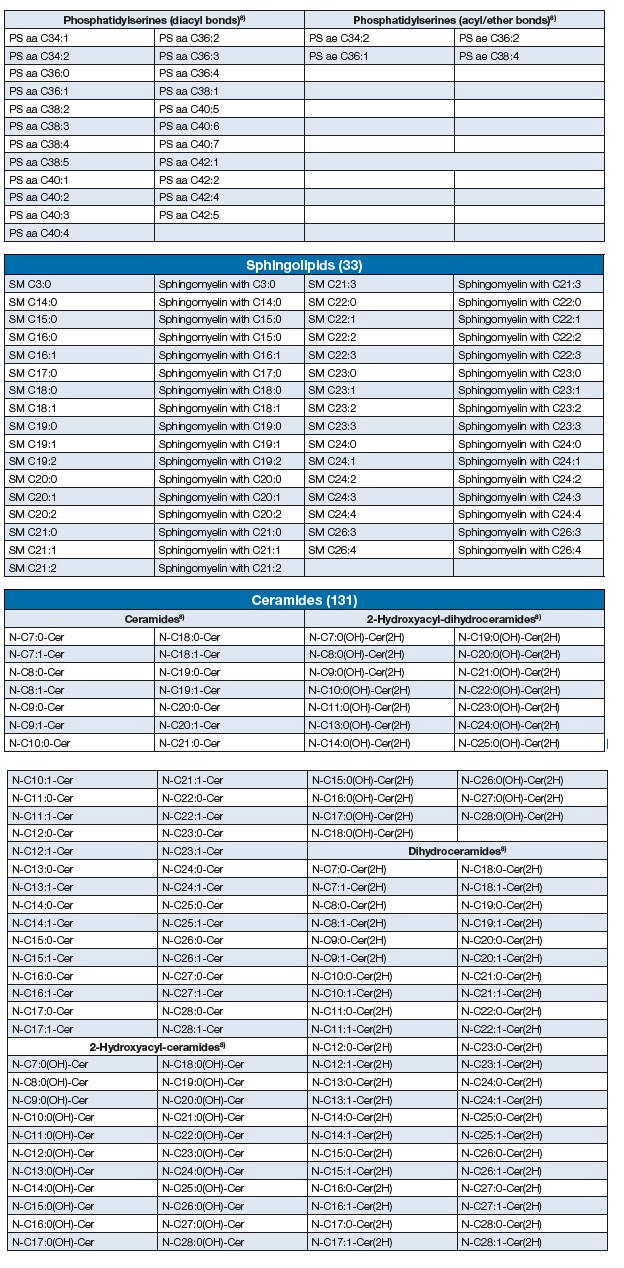
**
